# Spillover and crossover effects of exposure to work‐related aggression and adversities: A dyadic diary study

**DOI:** 10.1002/ab.22056

**Published:** 2022-10-25

**Authors:** Alexander Herrmann, Jürgen Glaser, Tobias Greitemeyer

**Affiliations:** ^1^ Institute of Psychology University of Innsbruck Innsbruck Austria

**Keywords:** adversities at work, crossover, dyadic data, spillover, workplace aggression

## Abstract

The past two decades have produced extensive evidence on the manifold and severe outcomes for victims of aggression exposure in the workplace. However, due to the dominating individual‐centered approach, most findings miss a social network perspective. Consequently, knowledge of negative spillover to different life‐domains or crossover to uninvolved individuals alongside a detailed understanding of the involved transmission processes remains scarce. By integrating social aggression theorizing, the present study investigated transmission routes (emphatic, behavioral) of experienced adversities and aggression at work toward perpetration of aggressive behavior and potential spillover and crossover effects into the private life domain in a diary study of 72 mixed dyads. Analyses of mediation based upon the Actor‐Partner Interdependence Model revealed an association between the frequency of perpetrating aggressive behavior in the work context and a spillover into the private life domain via aggression‐promoting internal states (emotions, cognitions, arousal). Based on the different patterns of mediation, it appears that adversities follow a mental transmission process, whereby experienced aggression displayed behavioral assimilation. In contrast, no crossover effects of exposure to adversities or aggression at work to a study partner at home could be detected. Practical and theoretical implications as well as limitations and ideas for future work are discussed.

## 1 INTRODUCTION

The frequency and severity of work‐related aggression has been found to be on the rise for decades (Chen, 2018; Douglas et al., 2008), posing as one of the greatest occupational hazards regarding employees' and organizational health (e.g., Gadegaard et al., 2018). Consequently, researching the negative effects of exposure to work‐related aggression and related phenomena has produced vast empiric evidence providing a detailed picture to its manifold and severe outcomes (e.g., Chang & Lyons, 2012; Flannery, 1996; Hershcovis & Reich, 2013; LeBlanc & Kelloway, 2002). However, previous research has predominantly been focusing on an individual‐centered perspective, neglecting that most victims of work‐related harmful behaviors are embedded in a social network, both in their professional as well as in their private lives. In other words, while it is well known what being exposed to harmful experiences at work can do to a victim, less is known about how such experiences spread across life domains and people socially surrounding victims (partners, coworkers, friends). The present research aims to fill this gap by illuminating how adversities and exposure to aggression at work evoke aggression in the worker, which then may spillover to aggression perpetration by the worker at home and those in close relationships with the worker.

### 1.1 The spillover−crossover model (SCM)

According to the SCM (Bakker et al., 2014), there are two transmission processes by which experiences from the work domain are carried over to the private life domain. First, *spillover* is defined as a “within‐person, across‐domains transmission of demands and consequent strain” (e.g., Bakker et al., 2014; p. 64), potentially leading to psychological and behavioral changes in other life domains. For example, stressful events at work might impair workers' detachment and recovery from work at the private domain in the evening. Second, *crossover* is defined as a transmission process between closely‐related individuals within the same life domain (e.g., Bakker et al., 2014). For example, workers' lack of detachment from work might cause conflicts with family members and impair their well‐being in the evening within the private domain. The SCM combines these two transmission routes to explain consequences of work characteristics for the private life domain, and occasionally vice versa. It is based upon the assumption that stress at work first spills over intra‐individually to employees' private life before crossing over to a partner through social interaction (Bakker et al., 2014). As stated above, this model has predominantly been used to explain effects of work‐related experiences toward indirectly related outcomes of a private life domain or person. In the present study, this model will be integrated into a framework to investigate a direct pathway of spillover and crossover effects whereby harmful experiences at work are associated with aggression‐promoting internal states and aggression perpetration in the work and private setting as well as toward closely related individuals.

#### 1.1.1 Aggression in previous spill‐ and crossover research

Thompson et al. (2017) identified 13 studies on the “dynamics” of workplace aggression in work and organizational research. These studies were primarily concerned with negative consequences for the family domain and vice versa, built mainly upon the stressor‐strain framework (Chen, 2018) as well as the conservation of resources theory (Hobfoll & Shirom, 1989, 2000). According to these theoretical perspectives, it was explained how a depletion of (limited) personal resources due to aspects of someone's work consequently leads to a reduction of resources to fulfill private life roles (spillover), decreasing for example, relationship satisfaction in a partner (crossover).

Experiences of harmful behaviors were found to cause distress (Demsky et al., 2014; Restubog et al., 2011) or negative affect in the victim at work (Hoobler & Hu, 2013), which negatively spills over to outcomes concerned with the victim's mental state or behavior when at home, “indirectly” assessed by outcomes such as work‐to‐family conflicts (WFC; Carlson et al., 2011; Liu et al., 2013; Wu et al., 2012), decreased family satisfaction (Carlson et al., 2011), or decreased marital/relationship satisfaction (Ferguson, 2012). Similarly, crossover effects were documented for stress transmission to the spouse (Ferguson, 2012), increased psychological distress of the partner (Haines et al., 2006), increased relationship tension (Carlson et al., 2011), or even partners' increased alcohol consumption (Marchand et al., 2009).

While those and similar findings are well in line with the overall SCM (Bakker et al., 2014), they fail to shine light on the underlying processes and transmission routes. To overcome this shortfall, work and organizational research could benefit from integrating evidence from social psychology that has conceptualized aggression as a “contagious disease” spreading through social networks (Bond & Bushman, 2017; p. 288). Precisely, recipients or targets of aggression were found to respond with enacted aggression themselves (e.g., Greitemeyer, 2019; Hershcovis & Reich, 2013), also toward innocent or uninvolved persons (Greitemeyer, 2018; Hoobler & Brass, 2006; Jung et al., 2019). Research in this field has further shown that if a participant's reference person (e.g., friend) has engaged in violent or aggressive behavior, the participant was more likely to engage in similar behavior, spreading from immediate friends to friends of friends (Bond & Bushman, 2017; Greitemeyer, 2018; Jung et al., 2019). The concept of “contagious disease” offers a more direct pathway of aggression leading to aggression toward others and ultimately causing relationship outcomes to decrease and WFC to increase.

#### 1.1.2 Work‐related aggression and adversities

When conceptualizing work‐related aggression, we follow the definition of Schat and Kelloway referring to “behavior by an individual or individuals within or outside an organization that is intended to physically or psychologically harm a worker or workers and occurs in a work‐related context” (2005, p. 191). To better understand how outcomes in the private life domain are negatively influenced by experiences of aggression at work, the present study aims to verify the two potential pathways, psychological/behavioral spillover as proposed in the SCM (Bakker et al., 2014) and empathic/emotional crossover (e.g., Westman, 2001; Westman et al., 2009) toward a “contagious spread” of harmful behaviors (Bond & Bushman, 2017; Jung et al., 2019). We do so because meta‐analytic evidence suggests that aggressive and violent stimuli (e.g., video games or movies) increase aggressive behavior as well as physiological arousal, aggression‐related thoughts and feelings (e.g., Greitemeyer & Mügge, 2014). This is well in line with the general aggression model (GAM; Anderson & Bushman, 2002) that provides a theoretical framework to assess such effects. Experiences of aggression at work are theorized to provide both learning and activation mechanisms of aggression‐promoting internal states (cognition, affect, arousal), which increase the likelihood for psychological and behavioral aggressive responses in the same environment (e.g., at work) and sequentially lead to assimilated psychological states and behaviors when in a different environment (e.g., at home).

So far, only few studies in work and organizational research have investigated these “direct” theoretical pathways. Two studies found similar behavioral‐based spill‐/crossovers via escalatory tendencies toward the spouse or coworkers (Winstok, 2006) and perpetrated aggression toward coworkers (Mitchell & Ambrose, 2012). While previous research found evidence for a psychological spillover looking at strain‐based family conflicts (Carlson et al., 2011; Liu et al., 2013; Wu et al., 2012) or depression (Lim & Lee, 2011; Miner et al., 2014) and an empathic crossover by examining stress transmission to the spouse (Ferguson, 2012), no study has yet investigated an empathic crossover for aggression (via cognition, emotion, negative arousal). In fact, work and organization research in this field has so far scarcely examined witnessed violent or aggressive behavior, which might also increase the likelihood for people to engage in similar behaviors (Bingenheimer et al., 2005; Patel et al., 2013).

**Figure 1:**
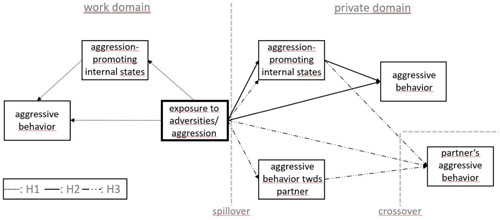
Conceptual model of hypotheses

Furthermore, we were interested in the question whether or not aggressive psychological or aggressive behavioral spill‐ and crossover effects will only be triggered by “high‐level” harmful behaviors. Consequently, the present study also includes low‐level stressors, such as adversities at work. Workplace adversity has been conceptualized as “any negative phenomenon or event experienced at work, or related to work, that makes it difficult for workers to perform and retain their jobs and to feel comfortable, satisfied, and happy in their place of work” (Vickers, 2016; p. 1). Adversities at work have also been found to potentially cause negative spillover and crossover effects (e.g., Leach & Butterworth, 2012). However, work‐related adversities are conceptually different from harmful behaviors such as aggression. They capture experiences of non‐harmful behavior of others, adverse circumstances, and situations that can cause feelings of frustration or anger in the recipient (e.g., Demsky et al., 2014), rather than aggression. Nevertheless, these “lower‐threshold experiences” of adversities at work might also be able to cause aggressive psychological or behavioral reactions. Based on these theoretical assumptions we postulate (see Figure 1):

#### 1.1.3 Adversities and aggression exposure evoking aggression perpetration

H1: *Exposure to adversities and aggression at work (direct and vicarious) increases aggressive behavior in the worker at work via aggression‐promoting internal states*.

#### 1.1.4 Spillover (psychological and/or behavioral)

H2: *Exposure to adversities and aggression at work (direct and vicarious) increases aggressive behavior in the worker at home via aggression‐promoting internal states at home*.

#### 1.1.5 Empathic and/or behavioral crossover

*H3: Exposure to adversities and aggression of the worker at work (direct and vicarious) increases aggressive behavior in the study partner at home via (a) the worker's increased aggression‐promoting internal states at home; (b) the worker's increased perpetration of aggression toward the study partner at home*.

The present study extends previous research in three key aspects by (1) investigating a direct contagious pathway of aggression, both direct and witnessed, via aggressive psychological and behavioral routes for both spillover as well as crossover effects; (2) integrating and testing social aggression theorizing (GAM; Anderson & Bushman, 2002) of non‐harmful but adverse experiences at work as additional causes for aggression‐promoting internal states or behavioral reactions of workers and their transmission to the nonwork domain to gain a more differentiated understanding of transmission processes captured by the SCM (Bakker et al., 2014); (3) including mixed dyad study partners, which takes upon the criticism that little research has been concerned with “nonfamily, nonwork domains” such as friends (rather than relationship partner or coworkers) when investigating crossover effects (Thompson et al., 2017; p. 208).

## 2 METHOD

General information as well as the invitation to the diary study was sent out through the university's student email distribution list providing a link to the online registration form. As part of the registration process students were asked to name a study partner, who would be willing to participate in the survey. As such, all workers were recruited from currently enrolled students (at least 20 h pers week), which posed as the minimum requirement in this study and perceived to have sufficient characteristics of a work environment. In other words, no none‐students or unemployed participants could take the study as workers (actors), while their study partners were not restricted by their “student/employment status” (partners). The study partner had to be closely related (daily contact) to the participants' private life (parent, intimate partner, friend, etc.) but not to their working life (fellow student, coworker, client, etc.). Apart from being a prominent part of the general participation conditions, this was verified through a respective item in the registration process of each member of a dyad asking about the kind of relationship between the participant and his or her study partner. Selection of a “work relationship” of one dyad member resulted in an incomplete registration process and the information that the study prerequisites were not met. This process led to *distinguishable* dyads in which one person was the worker and the other person was the study partner.

The study partner was then automatically contacted with information about the study asking individual consent for participation before providing a link to a similar registration form. After both participants had finished the registration process, their data sets were linked through individual numeric codes ensuring full anonymity. After a dyad had successfully passed this process, both participants received a daily “evening‐questionnaire” (at 7 p.m.) over the course of 7 consecutive days assessing their experiences in their working as well as their private life retrospectively for each particular day. Participants were instructed to complete each daily diary before going to bed. The study design was approved by the university's ethic board before data collection.

### 2.1 Sample

A total of 84 participants (workers) signed up for the study. An incomplete registration process (*N* = 5) and failing participation in the daily surveys (both participants: *N* = 5; one participant: *N* = 2), led to 72 complete dyadic data sets (144 participants) over the course of 2 months. The distinguishable dyads consist of 9 (12.5%) male−male, 22 (30.6%) female−female, and 41 (56.9%) mixed gender pairings with an average relationship duration of 10.1 years (SD: 8.5, Min: 0.17−Max: 29 years) and a shared household of 38 dyads (52.8%).

Workers were predominantly female (65.3%) with an average age of 23.4 years (SD: 6.5). The worker subsample comprised 50 students (69.4%) and 22 working students (30.6%) with actual weekly working hours of 33.1 h (SD: 13.0).

Study partners' gender was more balanced (52.8% female) with an average age of 28.4 years (SD: 13.1). The study partner subsample comprised 30 working individuals (41.7%), 16 students (22.2%), 11 working students (15.3%), and 15 people not working or studying (20.8%).

### 2.2 Measures

During the 7‐day‐diary process all of the following variables were recorded on a 5‐point‐scale (1 = *not at all*, 2 = *a little*, 3 = *fairly*, 4 = *substantial*, 5 = *extremely)* on a daily basis (evening) for workers and study partners.

Adversities at work were assessed using three self‐developed items capturing the organization of work, other people, external influences (“Today I was facing the following adversities at work. Problems with: (1) external influences [e.g., noise, weather]; (2) the organization of work [e.g., organizational processes and requirements]; (3) other people [e.g., social or communicative behavior, inequities]),” which were calculated into a single mean score to represent adversities at work.

Aggression at work was recorded via single items, asking for direct and vicarious exposure (e.g., “Today I was experiencing [witnessing] verbal/physical aggression by other people at work”) as well as perpetrating behavior (e.g., “Today I was displaying verbal/physical aggression toward other people at work”). Each of these aspects (experiencing, witnessing, perpetrating) of aggression were represented by 2 items, addressing verbal aggression (“yelling, swearing, raising the voice, making fun of someone, insulting someone, threatening someone”) or physical aggression (“insulting gestures, clenching one's fist, rolling one's eyes, banging on the table, pushing, punching”). Direct aggression at work (verbal and physical) together with vicarious aggression at work (verbal and physical) were calculated into a single mean score to represent exposure to aggression at work (victimization). Correspondingly, the extent of aggression perpetration at work was also averaged across the same verbal and physical aspects of aggressive behavior.

Aggression at home was assessed the same way as aggression at work but items additionally differentiated between perpetrators (other people vs. study partner) of the experienced behavior (victimization: “Today I was experiencing [witnessing] verbal/physical aggression by my study partner at home”; perpetration: “Today I was displaying verbal/physical aggression toward my study partner at home”). Participants were instructed to refer to their study partner, regardless if he or she was their intimate partner. Corresponding items were calculated into single mean scores representing experienced aggression at home by others, perpetration of aggression toward the study partner at home and general perpetration of aggressive behavior at home.

Aggression‐promoting internal states (at work vs. at home) were captured on three self‐developed items covering aggressive cognition (“I imagined to ignore someone, yell at someone, push someone, break something”), aggressive emotion (“I had the feeling to explode, felt rage, felt anger”), and negative arousal (“I felt negatively agitated, upset, stirred up”), which were calculated into a single mean score as measures for internal states at work and at home.

### 2.3 Data analysis

In the analysis of distinguishable dyadic data, experiences of a focal person are conceptually linked not only to individual outcomes but also to a study partner's (interdependence of data). If this interdependence in the data structure is not accounted for, biases in significance testing are known to occur (e.g., Kashy et al., 2018). Therefore, the Actor‐Partner Interdependence Model (APIM; Kashy & Kenny, 2000) and its extensions provide an established statistical standard when dealing with dyadic data, helping to control for the nonindependence of data in relationship dyads. However, the APIM involves a complex model structure when intensive longitudinal data (L‐APIM) is concerned. Due to restrictions of statistical power for the assessment of (too) many parameters in such a model, we could not apply a mediated L‐APIM. Missing data on work constructs due to nonwork days, low within‐subject variances and floor effects (see Table 1) provided additional challenges in the handling of the acquired data.

**Table 1:** Descriptive statistics and Pearson's correlation for study variables (worker *N* = 72, partner *N* = 72)

Variable	*M*	*SD*	1	2	3	4	5	6	7	8	9	10	11	12	13
1. Adversities at work/W	1.67	0.53	‐												
2. Adversities at work/P	1.56	0.57	.31**	‐											
3. Aggression at work/W	1.21	0.41	.59***	.41***	‐										
4. Aggression at work/P	1.25	0.40	.47***	.77***	.70***	‐									
5. Aggression‐promoting internal states at work/W	1.59	0.57	.74***	.33**	.50***	.45***	‐								
6. Aggression‐promoting internal states at work/P	1.53	0.68	.32**	.80***	.45***	.68***	.39***	‐							
7. Perpetration of aggression at work/W	1.15	0.35	.62***	.47***	.87***	.72***	.57***	.51***	‐						
8. Perpetration of aggression at work/P	1.20	0.39	.42***	.66***	.64***	.85***	.40***	.71***	.69***	‐					
9. Aggression‐promoting internal states at home/W	1.63	0.54	.57***	.14	.45***	.36**	.59***	.18	.42***	.36**	‐				
10. Aggression‐promoting internal states at home/P	1.61	0.49	.32**	.40***	.39***	.45***	.30*	.60***	.43***	.55***	.14	‐			
11. Perpetration of aggression toward partner at home/W	1.17	0.33	.39***	.23	.71***	.49***	.40***	.30**	.64***	.43***	.46***	.26*	‐		
12. Perpetration of aggression toward partner at home/P	1.24	0.33	.34**	.56***	.63***	.74***	.37**	.50***	.64***	.76***	.38**	.46***	.68***	‐	
13. Perpetration of aggression at home/W	1.20	0.31	.51***	.30*	.78***	.52***	.42***	.34**	.74***	.44***	.54***	.29*	.73***	.50***	‐
14. Perpetration of aggression at home/P	1.24	0.35	.29*	.51***	.56***	.70***	.28*	.55***	.57***	.78***	.32**	.60***	.51***	.76***	.49***

Abbreviations: P, partner; W, worker.

* *p* < .05

** *p* < .01

*** *p* < .001.

**Figure 2:**
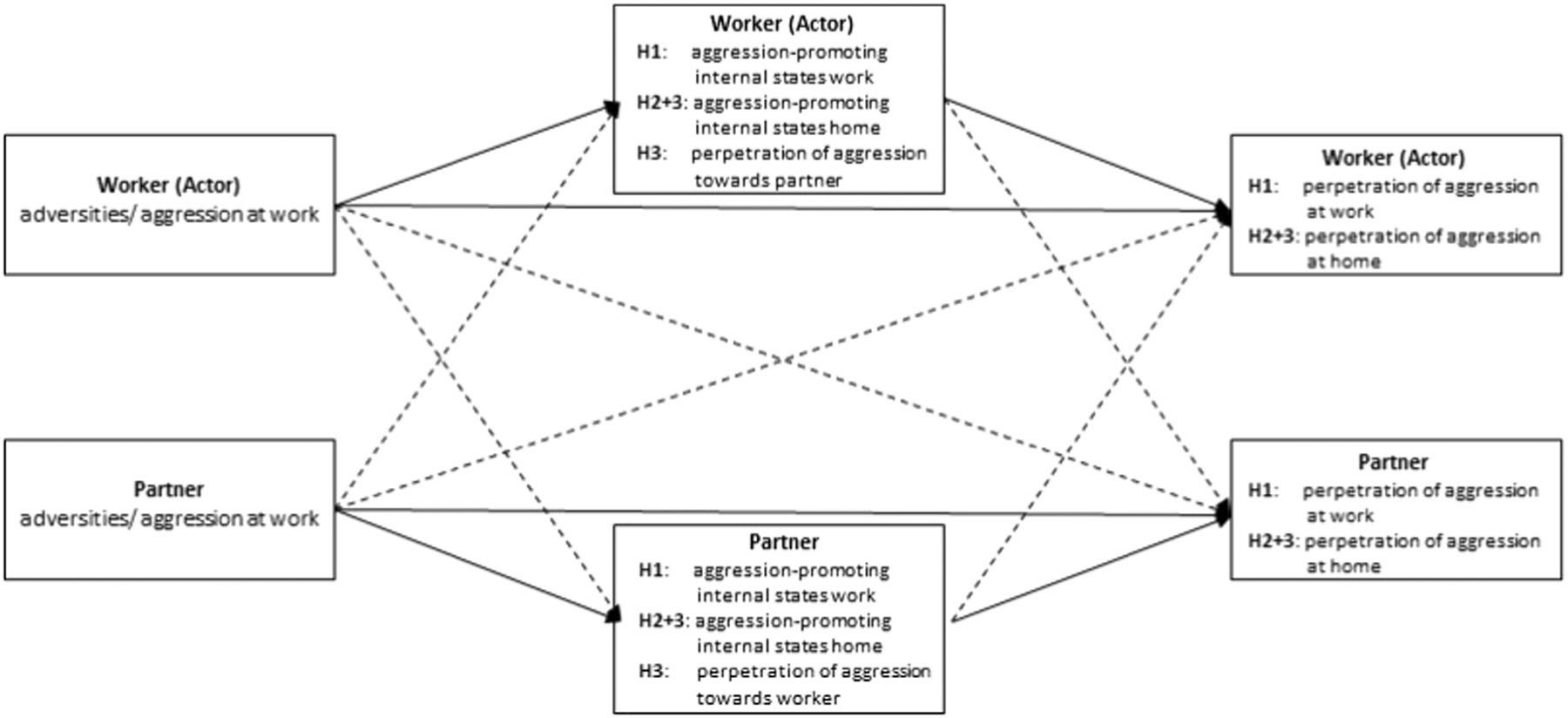
APIMeM model with hypotheses' related constructs. Solid lines represent common paths of H1 + 2, dotted lines represent crossover paths of H3. APIMeM, APIM extended to mediation.

To overcome these restraining factors but still be able to test our hypotheses in an adequate manner, we had to focus on within‐dyad effects based on a cross‐sectional data‐level. Therefore, longitudinal information was integrated into a mean score for each construct, representing the average score of each test person across our 7‐days assessment, which enabled the use of a standard APIM extended to mediation (APIMeM, Ledermann et al., 2011; see Figure 2). These “simpler” APIMeM models follow structural equation modeling and mediation analyses based on ordinary least squares regression (Coutts et al., 2019). Nevertheless, the way these underlying models are designed (see Figure 2), any putative outcome (or mediation variable), as they are measured identically for each member of the dyad, is acknowledged to consist of variance stemming from the actor (here, the worker) as well as the (study) partner (interdependence of data).

Hence, observed variation in any of the variables of interest will be statistically differentiated into an actor (worker) as well as a (study) partner effect (of influence to this variation). Depending on the hypothesis tested, one of those two effects is of focal interest while the other is being “controlled for.” For example, when testing for spillover effects (changes within the same person over changing environments), we were interested in the worker effect on the worker's outcome, while the study partner's influence on the same outcome (partner‐actor effect) is being treated similar to the influence of a covariate.

It is also important to note, that independently of the hypothesis tested (path of interest), APIM analyses always estimate and include all possible paths between the variables of the dyad members as illustrated in Figure 2, which makes it a very parameter intensive procedure, even at a cross‐sectional data level. The MEDYAD software for distinguishable dyads (SPSS 26; Coutts et al., 2019) was used to fit a model for each hypothesis (bootstrapping procedure 5000 samples). Since MEDYAD is restricted to one independent predictor, we tested each hypothesis by fitting independent models for exposure to adversities as well as aggression at work (victimization). All constructs were grand‐mean centered before analysis as suggested by Bolger and Laurenceau (2013).

## 3 RESULTS

First, inspection of bivariate correlations of the study variables (see Table 1) revealed substantial positive relations between all workers' (actors') and study partners' measures displaying expected dyadic interdependence, except for aggression‐promoting internal states at home. Second, all variables showed significant relationships in the expected direction. Precisely, exposure to adversities at work were positively linked to aggression‐promoting internal states at work and at home, perpetration of aggression at work and at home as well as toward the study partner at home (see Table 1).

Simultaneously, exposure to aggression at work (victimization) was positively linked to aggression‐promoting internal states at work and at home as well as perpetration of aggression at work, at home and toward the study partner at home (see Table 1). Furthermore, aggression‐promoting internal states at work were also positively linked to perpetration of aggression at work, aggression‐promoting internal states at home as well as perpetration of aggression at home and toward the study partner at home (see Table 1).

For detailed hypotheses testing in the APIM framework, we primarily report the most parsimonious model respectively to enhance readability and limit the report of random effects due to our sample restrictions. Nevertheless, we also computed and tested alternative models including relevant covariates (gender, age, weekly working hours, shared household).

The first APIM Model (APIMeM) for adversities at work (see Table 2) revealed a non‐mediated relationship between such exposure at work and aggressive behavior at work via aggression‐promoting internal states at work for the worker and a fully‐mediated process for the study partner. Exposure to aggression at work (victimization; see Table 2) also revealed a non‐mediated relationship with perpetrated aggressive behavior at work for the worker, while study partners showed a partial‐mediated process via aggression‐promoting internal states at work. Accordingly, Hypothesis 1 can be fully confirmed only within the study partners' subsample (consisting of a higher percentage of classic employment, as will be discussed later). Controlling for age, gender, and weekly working hours in alternative models did not significantly influence any of the reported relationships, except for workers' weekly working hours showing a positive influence on the direct actor effect between adversities and perpetration of aggression at work (*B* = 0.01, *SE* = 0.00, bCI: [0.00−0.01]).

**Table 2:** Total, direct, and indirect effects in the APIMeM (H1)

	*B*	*SE*	95% bCI
W: Exposure to adversities → W: perpetration of aggression at work (actor effect)			
Total effect	0.35	0.06	[0.23−0.47]
Direct effect	0.27	0.08	[0.11−0.44]
IE via aggression‐promoting internal states	0.06	0.02	[−0.07 to 0.20]
P: Exposure to adversities → P: perpetration of aggression at work (partner effect)			
Total effect	0.40	0.06	[0.27−0.52]
Direct effect	0.15	0.09	[−0.03 to 0.33]
IE via aggression‐promoting internal states	0.25	0.09	[0.06−0.40]
W: Exposure to aggression → W: perpetration of aggression at work (actor effect)			
Total effect	0.63	0.07	[0.49−0.76]
Direct effect	0.58	0.07	[0.44−0.72]
IE via aggression‐promoting internal states	0.05	0.03	[−0.00 to 13]
P: Exposure to aggression → P: perpetration aggression at work (partner effect)			
Total effect	0.75	0.08	[0.58−0.92]
Direct effect	0.58	0.10	[0.38−0.77]
IE via aggression‐promoting internal states	0.18	0.06	[0.08−0.32]

Abbreviations: APIMeM, APIM extended to mediation; bCI, bootstrapped confidence interval (5000 samples); IE, indirect effect; P, partner; W, worker.

The second APIM Model (APIMeM) for adversities at work (see Table 3) revealed a fully‐mediated relationship between exposure to adversities at work and aggressive behavior at home via aggression‐promoting internal states at home for the worker and a partial‐mediated process for the study partner. Exposure to aggression at work pointed toward a partial‐mediated relationship with aggressive behavior at home via aggression‐promoting internal states at home for the worker, while study partners showed a non‐mediated relationship (see Table 3). The partial mediation of workers turned into non‐mediation once controlling for weekly working hours (IE: *B* = 0.07, *SE* = 0.06, bCI: [−0.00 to 0.21]). Controlling for age, gender, weekly working hours as well as whether dyad members shared the same household in alternative models did not further significantly influence any of the other relationships reported. Accordingly, Hypothesis 2 (spillover) received full support concerning adversities but not aggression at work (non‐mediation). Additionally, results pointed toward an actor‐partner effect (crossover) for adversities at work on study partners' perpetration of aggression at home via workers' aggression‐promoting internal states at home (full mediation; see Table 3).

**Table 3:** Total, direct, and indirect effects in the APIMeM (H2)

	*B*	*SE*	95% bCI
W: Exposure to adversities → W: perpetration of aggression at home (actor effect)			
Total effect	0.27	0.06	[0.14−0.39]
Direct effect	0.12	0.07	[−0.02 to 0.27]
IE via aggression‐promoting internal states at home	0.13	0.07	[0.01−0.29]
IE via W: aggression‐promoting internal states on P: perpetration of aggression at home	0.11	0.06	[>0.00 to 0.23]
P: Exposure to adversities → P: perpetration of aggression at home (partner effect)			
Total effect	0.28	0.07	[0.15−0.41]
Direct effect	0.19	0.06	[0.07−0.31]
IE via aggression‐promoting internal states at home	0.10	0.04	[0.01−0.18]
W: Exposure to aggression → W: perpetration of aggression at home (actor effect)			
Total effect	0.62	0.08	[0.46−0.78]
Direct effect	0.55	0.08	[0.39−0.71]
IE via aggression‐promoting internal states at home	0.07	0.05	[>0.00 to 0.20]
P: Exposure to aggression → P: perpetration of aggression at home (partner effect)			
Total effect	0.52	0.10	[0.31−0.73]
Direct effect	0.41	0.10	[0.21−0.60]
IE via aggression‐promoting internal states at home	0.11	0.06	[−0.03 to 0.21]

Abbreviations: APIMeM, APIM extended to mediation; bCI, bootstrapped confidence interval (5000 samples); IE, indirect effect; P, partner; W, worker.

To test whether exposure to adversities and aggression (victimization) of a worker at work would be related to aggressive behavior in the study partner at home and if so, via aggression‐promoting internal states of the worker at home or perpetration of aggression toward the study partner at home (H3), we specified a parallel mediation APIMeM. As the MEDYAD software allows to estimate this rather complex model, this approach allows a direct comparison of these two potential paths (empathic crossover vs. behavioral crossover) while controlling for any common variances (Coutts et al., 2019). However, none of the paths displaying crossover effects from the worker or study partner to his/her dyad member reached statistical significance (see Table 4).

**Table 4:** Total, direct, and indirect effects in the two mediator APIMeM (H3)

	*B*	*SE*	95% bCI
W: Exposure to adversities → P: perpetration of aggression at home (actor‐partner effect)			
Total effect	0.10	0.07	[−0.04 to 0.24]
Direct effect	−0.06	0.06	[−0.18 to 0.06]
IE via W: aggression‐promoting internal states at home	0.04	0.04	[−0.04 to 0.11]
IE via W: perpetration of aggression toward the partner at home	0.07	0.05	[−0.03 to 0.17]
P: Exposure to adversities → W: perpetration of aggression at home (partner‐actor effect)			
Total effect	0.08	0.06	[−0.03 to 0.20]
Direct effect	0.09	0.05	[−0.02 to 0.19]
IE via P: aggression‐promoting internal states at home	0.01	0.02	[−0.02 to 0.06]
IE via P: perpetration of aggression toward the worker at home	0.04	0.06	[−0.03 to 0.19]
W: Exposure to aggression → P: perpetration of aggression at home (actor‐partner effect)			
Total effect	0.12	0.10	[−0.08 to 0.33]
Direct effect	−0.02	0.11	[−0.23 to 0.19]
IE via W: aggression‐promoting internal states at home	0.01	0.03	[−0.04 to 0.09]
IE via W: perpetration of aggression toward the partner at home	0.09	0.06	[−0.04 to 0.21]
P: Exposure to aggression → W: perpetration of aggression at home (partner‐actor effect)			
Total effect	−0.04	0.08	[−0.20 to0.12]
Direct effect	0.03	0.09	[−0.15 to 0.21]
IE via P: aggression‐promoting internal states at home	0.01	0.02	[−0.03 to0.07]
IE via P: perpetration of aggression toward the worker at home	−0.00	0.05	[−0.14 to 0.08]

Abbreviations: APIMeM, APIM extended to mediation; bCI, bootstrapped confidence interval (5000 samples); IE, indirect effect; P, partner; W, worker.

Including age, gender, weekly working hours as well as whether dyad members shared the same household in the models did not significantly influence any of the relationships reported. Consequently, analyses did not provide any evidence in support of hypothesis 3 (spillover to empathic vs. behavioral crossover).

## 4 DISCUSSION

Researching direct contagious pathways to spillover and crossover effects of work‐related adversities and exposure to aggression in a sample of privately related dyads revealed strong interdependence of study variables in the work and home domain (see Table 1). Even though interdependence is known to occur in dyads, this constitutes a valuable finding giving that previous evidence is mainly drawn from intimate partnerships (Thompson et al., 2017) rather than a wider range of relationships as in the current study (with only 52.8% sharing the same household). While people in intimate relationships are known to strongly influence each other in terms of psychological as well as behavioral reactions (e.g., Laursen, 2005; Lee et al., 2009), we also find levels of exposure to adversities and aggression at work, and thus work environments itself, to appear similar as well. While we can only assume that this finding might be linked to a similar social status or level of education, such potential effects of “socialized assimilation” might provide an interesting aspect for future research in this field.

Based on the SCM, we examined in our first hypothesis the consequences of exposure to adversities or aggression in a work environment. We predicted (and found) exposure to adversities or aggression at work to be related to perpetration of aggressive behavior in a worker via his or her aggression‐promoting internal states (emotion, cognition, negative arousal). However, while all participants showed a significantly positive relationship between exposures and perpetrating behavior at work, workers and study partners differed with respect to the mediation of these effects. While study partners' experiences of adversities and aggression seem to be associated with aggression‐promoting internal states that in turn were related to aggressive behavior, workers seemed to behave more aggressively regardless of their internal states (cf. Table 2).

Nevertheless, our analyses clearly suggest that both phenomena are associated with aggressive behavior in the recipient, while adversities might follow an indirect route: initially via aggression‐promoting internal states due to caused frustration (frustration‐aggression theory, Berkowitz, 1989; Dollard et al., 1939). In turn, exposure to aggression (victimization) appears to have a direct statistical effect on aggressive behavior (and aggression related internal states), which could be explained as a result of social learning and assimilation/imitation processes (Anderson & Bushman, 2002; Bandura, 1977, 1986).

We also tested if those transmission routes would be evident with respect to a spillover into the private life domain. In fact, our analyses show that adversities and aggression at work are both related to aggressive behavior at home (spillover), while also following the same patterns of transmission routes (see Table 3). Again, adversities at work appear to be mediated through aggression‐promoting internal states (worker: full‐mediation vs. study partner: partial mediation), while exposure to aggression tends to show a direct (behavioral) association (worker: partial‐mediation vs. study partner: no mediation). Precisely, being exposed to adversities at work will be carried over to someone's private life via related emotions, cognitions, and arousal, as workers might fail to psychologically detach from work experiences and leave work‐related states at their doorsteps (Demsky et al., 2014). The experience of aggressive behaviors at work however, seems to promote reenactment of behaviors that people have been witnessed or were engaged in while at work as they are not (or to a lesser extent) mediated by aggression‐promoting internal states (Anderson & Bushman, 2002; Bandura, 1977, 1986).

Lastly, we investigated if spillover effects of exposures at work to the private domain would also crossover to the study partner. Since theory suggests crossover to occur via empathic and behavioral interaction between individuals (Bakker et al., 2014; Westman, 2001; Westman et al., 2009) we used aggression‐promoting internal states and perpetration of aggressive behavior toward the study partner as mediators in a parallel mediation model (APIMeM; Coutts et al., 2019). Although primary analyses (see hypothesis 2, Table 3) point toward an empathic crossover to study partners' increased aggressive behavior via workers' increased aggression‐promoting internal states, the parallel mediation model did not show any statistically significant paths to verify a crossover of any kind in our sample (see Table 4). Even though exposures at work show a positive relationship with aggression‐promoting internal states and perpetration of aggressive behavior toward the study partner at home (see Table 1), the influence appears not to be strong enough to be able to cross over to the study partner, neither empathically nor behaviorally.

We will now discuss several limitations that may have contributed to why there was no crossover effect. Floor effects and little variance within the exposure levels to adversities of our sample indicate that we did not collect data of highly exposed individuals as in high risk professions. In this context, it must be noted that a substantial part of the sample consisted of (working) students and pandemic prevention measures might have reduced exposures at work at this time (home office, less face‐to‐face interactions). Additionally, due to these limitations we were unable to estimate more complex longitudinal models and within‐dyad effects that might have provided a finer mashed approach to capture interactive processes and changes over time, which also must be noted for the interpretation of causality. Moreover, intraindividual (spillover) effects might be inflated by common method bias (Podsakoff et al., 2003).

We hope that future studies can overcome these limitations with larger and more suitable samples to replicate and extend the findings presented here. Investigating privately related dyads other than dual earner couples or intimate relationships is important to extend existing knowledge beyond those specific actor‐partner constellations. However, mixed dyads potentially complicate the opportunities to detect crossover effects in smaller samples, especially in terms of evidence for empathic transmission routes. Correspondingly, larger samples are also needed to be able to investigate specifics of privately related dyads based on their different relationships (friends, siblings, children‐parents, etc.). While daily contact within dyads was a mandatory prerequisite when enrolling in the study, a corresponding variable recording the extent of actual contact over the course of the study was not administered. We advise future studies to include such a covariate when investigating crossover effects.

Furthermore, not having dual earner couples might have contributed to the noted floor effects as workers not encountering any exposures received the same value coding as participants not working (and thus not being exposed). As estimation of an APIM model (Kashy & Kenny, 2000) requires to have the same measures for actors (workers) and (study) partners simultaneously and MEDYAD software only supports pairwise deletion of missing data (Coutts et al., 2019), not differentiating between nonworkers and nonexposed individuals prevented the loss of further data sets in the analyses. Future research should overcome this limitation by having employment as a prerequisite for both members of a mixed dyad or use different statistical approaches including data imputation.

Finally, some of our measures might require improvements in future research. We followed recommendations to include (less harmful) adversities at work which have been shown to be determinants for experiences of aggression at the workplace (Demsky et al., 2014). Due to time restrictions of diary studies, we could only operationalize three categories of potential adversities at work (i.e., problems with work environment, organization of work, and interaction with other people at work) by means of single items with rather diverse examples for each category. Future studies might use more fine‐grained instruments to examine these and other possible adversities at work in a more systematic manner. Furthermore, our items measuring physical aggression included examples of physically harming behavior like pushing or hitting others, but also insulting or aggressive gestures toward others. Future research might differentiate more precisely between physical aggression (in a narrower sense) and postural aggression.

## 5 CONCLUSION

Based on the SCM, this study aimed to shed more light on the contagious spreading of exposure to aggression (including witnessed aggression) or aggression inducing but non‐harmful exposures to adversities at work into perpetration of aggression. A better understanding of such mechanisms is crucial to provide potential avenues when designing interventions to tackle workplace aggression and its consequences. This study breaks new ground by employing dyadic data suggesting that aggressive and adverse experiences “use” different psychological (via associated internal states of cognition, emotion, or arousal; e.g., GAM, Anderson & Bushman, 2002) and behavioral routes (via social learning/assimilation; Bandura, 1977, 1986) as theorized by social aggression modeling when spreading across different life domains. The theoretical model presented here may inspire future empirical work toward a deepened understanding of how adversities and aggression spread between work and private life.

## CONFLICT OF INTEREST

The authors declare no conflict of interest.

## Data Availability

The data that support the findings of this study are available from the corresponding author upon reasonable request.
